# Gradual reputation dynamics evolve and sustain cooperation in indirect reciprocity

**DOI:** 10.1371/journal.pone.0329742

**Published:** 2025-08-08

**Authors:** Hitoshi Yamamoto, Isamu Okada, Takahisa Suzuki

**Affiliations:** 1 Department of Business Administration, Rissho University, Tokyo, Japan; 2 Department of Business Administration, Soka University, Tokyo, Japan; 3 College of Policy Studies, Tsuda University, Tokyo, Japan; Teesside University, UNITED KINGDOM OF GREAT BRITAIN AND NORTHERN IRELAND

## Abstract

Humans have achieved widespread cooperation, largely sustained by mechanisms such as indirect reciprocity, which relies on reputation and social norms. People are highly motivated to maintain a good reputation, and social norms play a critical role in reputation systems by defining acceptable behavior, helping prevent exploitation by free-riders. However, there is a gap between theory and experiment in handling reputation information, with experiments often failing to capture the complexity that theoretical models propose. Here, we address two key issues: what kind of information is needed to define reputation as a social norm and the appropriate level of granularity required for reputation information to function effectively. This paper combines scenario-based experiments and evolutionary game theory to investigate the social norms individuals adopt in real-world settings, aiming to uncover the stability of these norms. Our results show that reputations should be categorized into three levels good, neutral, and bad. Results suggest gradual reputation dynamics that increase and decrease gradually due to cooperation or defection. However, a person’s reputation remains unchanged only when they defect against a bad reputation. Our experimental and theoretical results support critical insights into the dynamics of reputation and social norms within indirect reciprocity, challenging traditional binary reputational evaluations. The gradual nature of reputation updating and the use of nuanced evaluations provide a more realistic model of reputation dynamics.

## Introduction

While various species exhibit cooperative behaviors [1, 2], no species has achieved the same level of widespread cooperation as humans [3]. Among the mechanisms identified for the evolution of cooperation [4], indirect reciprocity [5, 6] stands out as a particularly powerful factor in sustaining cooperation within large and dynamic societies. Indirect reciprocity, where individuals help others and receive help in return from different individuals, hinges on the crucial elements of reputation and the social norms that govern it.

Social norms, which define what is considered “good” or “bad” behavior, are integral to the functioning of reputational systems. These norms help individuals discern who deserves cooperation and who does not, thereby preventing exploitation by free-riders. Extensive research across various fields, including biology, physics, economics, and psychology, has sought to identify which norms can sustain a stable cooperative society. For instance, norms such as “cooperation is good and defection is bad” [7, 8], “cooperating with a bad person is bad, and defecting against a bad person is good” [9–12], and “even cooperating with a bad person is good, and defecting against a bad person is also good” [5, 13, 14] have been extensively studied.

In this paper, we address two key issues in indirect reciprocity. The first concerns the role of higher-order information, which involves the complexity of reputation information used to evaluate social interactions. The second focuses on the granularity of reputation information, specifically how detailed or simplified reputation categories need to be for effective social norms to emerge.

The importance of higher-order information has been emphasized in theoretical studies, which have proposed complex models that consider not only first-order information (the actor’s behavior) but also second-order (the recipient’s reputation) and even third-order information (the actor’s reputation). These models suggest that cooperation becomes more robust as higher-order information is incorporated [12, 15–17]. However, there is a significant gap between theory and experiment: while theoretical studies highlight the potential benefits of higher-order information, experimental studies often neglect these complexities. Some experimental findings suggest that humans prefer simpler, first-order information due to cognitive limitations [18], while others indicate that individuals are willing to use higher-order information despite its complexity and associated costs [19, 20]. However, existing studies have primarily focused on whether second-order information is used, without examining in detail how individuals incorporate such information into their reputation evaluations and decision-making processes. The gap underscores the need to further investigate how humans evaluate and utilize higher-order information in practice, particularly in scenarios where cooperation depends on such nuanced assessments.

The granularity of reputation information refers to the degree of detail or resolution in the categories used to represent reputation. While theoretical models often simplify reputation into binary categories (e.g., good or bad) for analytical tractability [15], real-world interactions suggest a more nuanced approach is necessary. For example, recent studies indicate that people may use multi-valued reputations, such as including a “neutral” category alongside “good” and “bad” [21]. Despite this, experimental research on the granularity of reputation information remains limited, with few studies providing empirical evidence to determine the appropriate level of granularity that can inform theoretical models.

Moreover, from a methodological perspective, there is a significant gap between experimental and theoretical approaches. Although extensive literature has examined indirect reciprocity, most studies have employed experimental [18, 22–26] or theoretical [14–16, 27–30] approaches independently. Experimental studies often focus on how people evaluate others’ actions and assign reputations, yet these evaluations are not always grounded in theoretical analysis, making their evolutionary stability difficult to assess. Conversely, theoretical studies comprehensively analyze cooperation’s evolutionary stability but typically neglect the applicability of these findings to real-world behaviors. This disconnection limits our understanding of how social norms function and persist in human societies.

In this paper, we address these gaps by combining experimental and theoretical approaches to investigate the social norms that individuals adopt and their evolutionary stability. To do so, first we conduct a scenario-based experiment under which reputation-determining information includes both the actor’s reputation (third-order information) and the recipient’s reputation (second-order information), with all reputation information being three-valued, i.e., good, neutral, and bad. This experiment is to investigate the social norms individuals adopt in real-world contexts, and the experimental scenarios systematically vary the actor’s behavior (cooperative or defective), the reputation of recipients (good, neutral, or bad), and the reputation of actors (good, neutral, or bad). After clarifying social norms employed in real-world contexts, we rigorously assess their evolutionary stability using mathematical analysis. We will analyze replicator dynamics using the framework of evolutionary game theory to determine whether the norm discovered in the experiment is evolutionarily stable. By combining these approaches, we aim to identify the social norms prevalent in real societies and provide a comprehensive understanding of why these norms emerge and persist.

## Results

### Experimental exploration of social norm

We designed a scenario-based experiment to elucidate how individuals apply evaluation rules in indirect reciprocity scenarios. The scenario involved two characters: one as the donor, who decided whether to cooperate with the recipient, and the other as the recipient, who requested cooperation from the donor. We characterized the donor and recipient through prior information for three types of reputation: “good reputation,” “bad reputation,” and “neutral (no reputation information).” In this study, we conducted an experiment in which neutral reputation conditions were represented by the absence of reputation information. However, the absence of reputation information does not necessarily equate to a neutral reputation. To determine whether having no reputation information corresponds to having a neutral reputation, we analyzed participants’ evaluations of recipients without reputation information. The results indicated that participants assigned neutral evaluations to recipients without reputation information (S2 Fig). The experiment featured 18 different scenarios, combining variations in donor behavior (cooperate or not), donor reputation (good, bad, or neutral), and recipient reputation (good, bad, or neutral), and it was executed using a between-subjects design. Our experimental setting builds upon the previous research [21]. Previous research analyzed four specific cases in which a donor with a neutral reputation either cooperates with or defects against a recipient with a good or bad reputation. Participants were randomly assigned to one of 18 scenarios and gave their impressions of the donor’s behavior.

**Fig 1:**
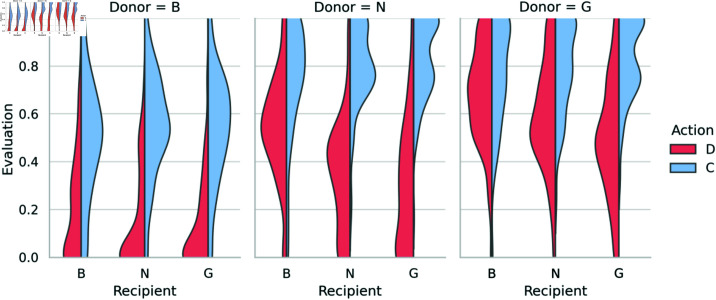
Distribution of evaluations of donor’s behavior: Violin plots show distribution of donors’ behavior evaluations for each of 18 scenes. Red and blue correspond to defection and cooperation, respectively.

The experimental results indicate that all 18 scenes can be categorized into three types of distributions: a right-skewed distribution (evaluated as bad), a left-skewed distribution (evaluated as good), and a distribution with a central peak (evaluated as neutral) (Fig 1). First, we conducted a cluster analysis using the Wasserstein distance to classify participants’ evaluations of the 18 scenes, resulting in three distinct clusters (S1 Fig). Next, to identify the characteristics of each cluster, we fitted three distributions, a normal distribution (interpreted as neutral), an exponential distribution (interpreted as bad), and an exponentially reversed distribution (interpreted as good), using the maximum likelihood estimation method. We determined the best-fitting distribution for each distribution using the Bayesian Information Criterion (BIC). The results (Table S2) indicated that the clusters could be characterized as a right-skewed distribution (bad), a left-skewed distribution (good), and a distribution with a central peak (neutral). Table 1 presents the classification results for each scene, indicating that the reputation values were classified as “good”, “bad”, and “neutral”. These findings reveal that irrespective of the donor’s reputation, cooperative behavior consistently improved the rating by one level in a positive direction. In contrast, defection generally resulted in a downgrade of one level in the evaluation. However, defection against bad recipients was an exception, as their reputation remained unchanged. In other words, when a neutral donor engaged in justified defection, their reputation stayed neutral, whereas justified defection by a good donor was rated as good. These results suggest that justified defection is only acceptable when carried out by donors with a good reputation.

**Table 1:** Evaluation rule considering third-order information and multiple reputation values: By cluster analysis and best-fitting distribution for each distribution done on basis of BIC, reputation values are classified as good, bad, and neutral.

Action (C/D)	D									C								
Who (Donor)	B			N			G			B			N			G		
To whom (Recipient)	B	N	G	B	N	G	B	N	G	B	N	G	B	N	G	B	N	G
Evaluation	**B**	**B**	**B**	**N**	**B**	**B**	**G**	**N**	**N**	**N**	**N**	**N**	**G**	**G**	**G**	**G**	**G**	**G**

**Fig 2:**
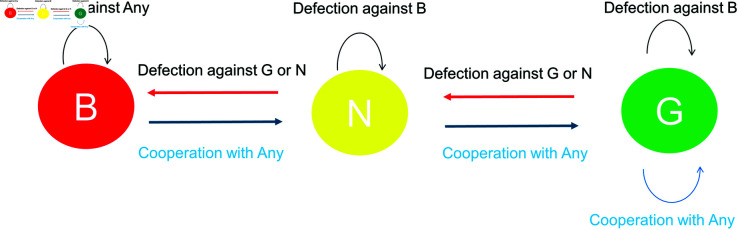
State transition dynamics of reputation considering third-order information and multiple reputation values: Reputation improves by one step for cooperation and deteriorates by one step for defection. However, when defection (justified defection) occurs against bad recipient, donor’s reputation remains unchanged.

The results in Table 1 allow us to construct a state transition diagram that represents the dynamics of reputation (Fig 2). Participants exhibit three types of reputation towards others, good (G), neutral (N), and bad (B), and these reputations are evaluated by one point on the basis of observed behavior. Generally, cooperation and defection are evaluated as positive and negative, respectively. However, contrary to prior research assumptions, individuals apply a more moderate reputation updating rule. Specifically, defection against a bad donor does not alter the reputation. Justified defection is considered acceptable only for donors with a good reputation. Reputation updating occurs gradually, with no dramatic shifts, such as bad becoming good or good becoming bad. We refer to the social norm revealed by this experiment as “gradating”. This norm is characterized by gradated reputation updates and a neutral attitude towards justified defection, where reputation remains unchanged. These features can be viewed as an extension of the L1 norm from the “leading eight,” [15] applied to a multi-valued reputation system.

Overall, the results suggest that reputation updates in social interactions are gradual. Justified defection is conditionally accepted on the basis of the donor’s given reputation, highlighting the importance of a given reputation status in evaluating subsequent actions. Although the cooperation of a donor with a good reputation is naturally rated as good, the five-point scale used in this experiment makes it unclear whether the evaluation has improved or simply remained good. The same applies to the defection of donors with a bad reputation. Theoretical research has explored various levels of reputational granularity [7, 31], but the appropriate granularity of reputations remains controversial in experimental research. Future research should address whether reputation should be expressed using three values or extended to a more nuanced scale.

Our experimental setting can be regarded as private in that observers independently evaluate the donor, while public in that the donor and recipient’s reputation are presented as shared information. The analytical model employs a public information structure. On the other hand, recent studies of indirect reciprocity have extensively employed private assessment models [28, 29, 32–34]. The results of this study align with findings from private evaluation research, demonstrating that cooperation is consistently evaluated positively even when reputation is public. Future work should examine whether similar patterns emerge in experiments and simulations based on fully private assessment systems.

### Theoretical analysis of social norm

We will examine whether the “gradating” social norm discovered in our experimental study can be accepted from a theoretical point of view. To do so, we consider evolutionary game theoretical analysis using replicator dynamics, to look for adaptive action rules keeping cooperative regimes, assuming that all players adopt the social norm discovered.

First, we model the gradating social norm. The gradating uses a ternary reputation label, so we define a set of labels as R={0,1,2} where a reputation label {0,1,2} corresponds to bad, neutral, and good, respectively. Additionally, any action is binary, so we define that an action is 1 when one cooperates and that an action is 0 when one defects. In a social norm using a ternary reputation label, the number of configuration types of action rules is eight: $A=\{a_{0},a_{1},\ldots ,a_{7}\}$. The numbering of those eight types is denoted as a three-letter string. For example, action rule *a*_3_ transforms its suffix to [011] by following a binary transformation. The meanings of each letter are as follows. The first letter is an action to someone with a 0 reputation label. The second and third letter are, respectively, an action to someone with a 1 and 2 reputation label. For example, if a player uses the action rule *a*_3_, the player cooperates with someone who has either a 1 or 2 reputation label only. Note that an unconditional cooperator corresponds to a player who adopts *a*_7_ while an unconditional defector corresponds to an *a*_0_ player.

Here, we model the evolutionary game for infinite well-mixed players. Let population $p=(p_{0},p_{1},\ldots ,p_{7})$ be the state in which the proportion of players adopting action rule *a*_*i*_ is *p*_*i*_, where $\sum_{i}p_{i}=1$, and $0\leq p_{i}\leq 1$. To explore the evolutionary dynamics of the reputation labels, we assume that the time scale for natural selection is much slower than that for social interactions and label updating [35]. Therefore, we can assume that the frequency of labels is always at an equilibrium value, i.e., the expected probability of a player’s reputation label converges to a steady state. Let $g_{r}(a_{i}|p)$ be the fraction of players with action rule *a*_*i*_ having an *r* reputation label in population *p*, where $\sum_{r}g_{r}(a_{i}|p)=1$, and $0\leq g_{r}(a_{i}|p)\leq 1$ for any r∈R and $a_{i}\in A$. The system of equations for computing the equilibrium values of $g_{r}(a_{i}|p)$ and the results are shown in Methods.

To explore adaptive action rules, all players change their own action rules following an evolutionary process. Replicator dynamics [36] models the natural assumption that players who obtain higher-than-average (expected) payoffs are more likely to increase their proportion, and it is suitable for exploring adaptive strategies through natural selection in biology and other fields. In addition to natural selection through replicator dynamics, here we consider a more realistic analysis by analyzing replicator dynamics with perturbations [37] that models the random entry of free strategies as a small percentage of mutations.

We introduce two parameters to generalize the model and to consider a real situation. One is an implementation error, in which there is a probability *e* of not cooperating when a player intends to cooperate where 0≤e≪1. To calculate their expected payoffs, we use two game parameters: cost of cooperation (*c*) and benefit to its recipients (*b*) where *b*>*c*>0. We are ready to define an expected payoff for a player using action rule *a*_*i*_ in population $p=(p_{0},p_{1},\ldots ,p_{7})$,

$$U_{i}(p)=(1-e)b\sum_{a_{j}\in A}p_{j}\sum_{r\in R}g_{r}(a_{i}|p)j_{r}-(1-e)c\sum_{a_{j}\in A}p_{j}\sum_{r\in R}g_{r}(a_{j}|p)i_{r}$$ (1)

where $\left[i_{0}i_{1}i_{2}\right]$ and $\left[j_{0}j_{1}j_{2}\right]$ are binary transformations of *i* and *j*, respectively, i.e., $i=4i_{0}+2i_{1}+i_{2}$ and $j=4j_{0}+2j_{1}+j_{2}$.

In this system, for a population ( = *p*) consisting of eight types of action rules *A* = {*a*_*i*_}, the proportion of reputation labels of each action rule strategist $g_{r}(a_{i}|p)$ is included as factors that constitute the payoffs of each action rule, so it is extremely difficult to calculate an analytically exact solution and determine which action rule is dominant for each population. However, what is important in the question of whether the social norm is theoretically possible is to examine the possibility of a stable population existing that can maintain a high cooperation rate. Therefore, we first consider the evolutionary stability (ESS) for a single population, that is, a population in which everyone adopts the same single action rule. As shown in Methods, the analysis when there are no errors (that is, when *e* = 0) revealed that *a*_2_ is ESS. However, because the cooperation rate of the group consisting of *a*_2_ only reaches about one-third, this is not a desirable social norm in the sense that perfect cooperation is achieved. A more important problem is that the action rule *a*_2_ is generally difficult to understand. In other words, this action rule is denoted as [010], and players choose to cooperate only with others whose reputation rule is 1 (neutral) and not cooperate with others whose reputation is good or bad. Note that, however, there are possible interpretations that can justify such action rules. For example, if a player has a good reputation, she or he may choose not to cooperate because her or his reputation will not be damaged immediately.

To overcome this point, the action rule needs to be extended to a more realistic setting. Thus, in addition to introducing errors, the action rule is formulated from the viewpoint that it is always exposed to the opportunity for new entrants. Therefore, we introduce the mutation rate *μ* as a second parameter, in which there is a probability *μ* of random players invading the population for the replicator dynamics with perturbations where 0≤μ≪1. The frequency of players with action rule $a_{i}\in A$ in the population *p* changes over time in accordance with the replicator dynamics with perturbations as follows:

$$\dot{p_{i}}=p_{i}(U_{i}(p)-\bar{U}-\mu )+\frac{\mu }{\left|A\right|}$$ (2)

where $\bar{U}=\sum_{i}p_{i}U_{i}(p)$ and each action rule increases the quantity of μ/|A| as a mutant in any period, and therefore, each should decrease the quantity of $\mu p_{i}$ in order to replace the mutants.

**Fig 3:**
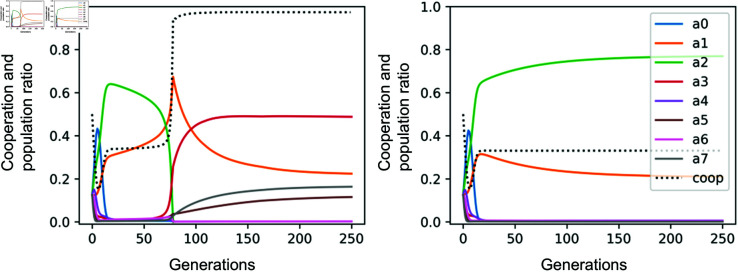
Replicator dynamics on action rules and cooperation rate Each panel represents generational consequences of changing the fraction of all action rules $A=a_{0},a_{1},\ldots ,a_{7}$ and the cooperation rate. The horizontal axis represents generations that update strategies. Parameters are set to (b,c)=(3,1) and (e,μ) is Left: (2%,2%), and Right: (5%,1%). The initial population is set to p=(1/8,1/8,…,1/8). The dotted black line shows the cooperation ratio, and the solid colored lines show the population ratio of each action rules.

We are ready to analyze the model using numerical simulations. Fig 3 shows the time-series performance and cooperation rate of the action rules using the replicator dynamics with perturbations. From the left panel, we can see that the dynamics has four time phases. First, *a*_0_ or unconditional defectors gain power and drive many action strategies (especially *a*_4_ to *a*_7_) to extinction. Note that our dynamics model is perturbed so that a certain number of mutations can always be introduced, so no strategy is completely extinct. Next, *a*_2_, an odd action strategy that cooperates only with neutrals, forms the majority. At this time, the cooperation rate slowly rises to about 1/3. This is because this action strategy, if it forms a single population, will share the reputation information of good, neutral, and bad equally, with 1/3 (See the “fractions of reputation labels with each action rule” in Methods for details). In the second phase, *a*_1_, which is a strict rule that only cooperates with good, can invade because it has the same expected payoff (See the “ESS analysis in single population with (e,μ)=(0,0)” in Methods for details). When the population of *a*_1_ eventually exceeds that of *a*_2_, the short third phase begins. At this time, *a*_1_ suddenly becomes the majority, and *a*_2_ is driven to extinction. In this third phase, the *a*_3_ strategy, which is dominant over *a*_1_, rapidly increases the population, and the cooperation rate rises to nearly 100%. Then, in the final phase, the *a*_3_ strategy forms the majority, and the society becomes stable as a cooperative society. The *a*_3_ strategy can be said to be a tolerant rule in prosocial behavior because it cooperates with good and neutral.

**Fig 4:**
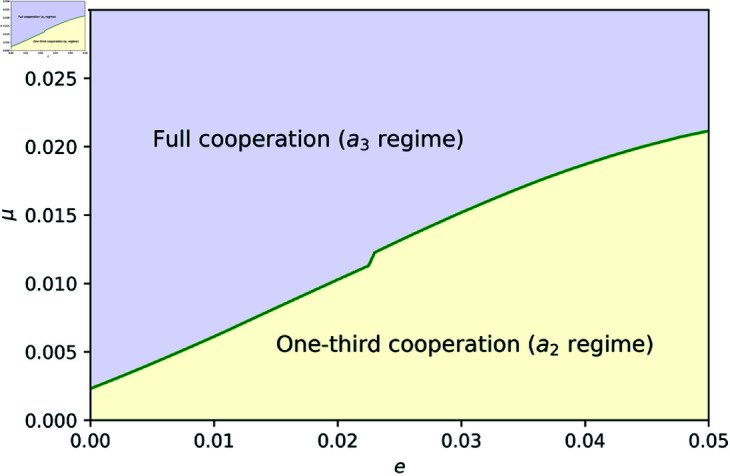
Action rules that forms the majority in equilibrium: As shown in Fig 3, the equilibrium state depends on the model parameters, particularly the error rate (*e*) and the mutation rate (*μ*). The system converges either to the *a*_3_ regime, where the cooperation rate is nearly full and *a*_3_ dominates the population (left panel of Fig 3), or to the *a*_2_ regime, where the cooperation rate is approximately one-third and *a*_2_ is most frequent (right panel of Fig 3). The figure indicates which regime emerges at equilibrium across different combinations of (e,μ) values, with yellow representing the *a*_2_ regime and purple representing the *a*_3_ regime. The parameters are set as (b,c)=(3,1).

Both panels of Fig 3 show the performances with different parameters, and except for the slower phase transition, it basically has four phases, just like the left panel. In the right panel of Fig 3, only up to the second phase occurs. This is because when *a*_2_ is the majority, *a*_1_ can invade if the population ratio of *a*_2_ is not too high. In the right panel of Fig 3, the population ratio of *a*_2_ is too high, so the system converges before the third phase. In other words, depending on the parameters, there are two patterns: up to the second phase, where *a*_2_ converges with the majority, and reaching the fourth phase, where *a*_3_ converges with the majority. The point here is that when *a*_3_ becomes the majority, the cooperation rate can reach almost 100%, whereas when *a*_2_ becomes the majority, the cooperation rate remains at about 1/3. In order for the system to maintain cooperative regimes, *a*_3_ needs to form the majority. Fig 4 shows which rules will become the majority depending on the parameters of *e* and *μ*. While the results shown in Results simulate a dynamics from a specific initial population, the results are quite robust to the composition of the initial population, as shown in Methods.

## Discussion

In his famous *Othello*, premiering in 1604, Shakespeare made Cassio say, “Reputation, reputation, reputation! O, I have lost my reputation!” As depicted in famous classics, people are greedy in acquiring a good reputation and feel great pain over losing a good reputation [38]. It is well recognized that a significant portion of human communication centers on the exchange of reputational information [39, 40]. Reputation undeniably serves as a fundamental pillar of human society, yet research on reputation dynamics has often lacked a cohesive integration of theory and experimentation. Here, we clarify the dynamics of reputation that individuals adopt through experimental investigations, aiming to construct a theoretical model strongly supported by experimental evidence.

We designed an experimental and theoretical framework that considers both higher-order information and a multi-value reputation. Our experiment categorized all cases containing third-order information into three reputation levels: good, bad, and neutral. The experimental results show that cooperative behavior always increases the donor’s reputation by one level, while defection generally decreases it by one level. However, defection against a bad recipient does not alter the donor’s reputation. Justified defection maintains the donor’s reputation, and if performed by someone with a good reputation, it continues to be evaluated positively. These findings suggest that reputation updates are gradual and that evaluations of cooperation and defection are influenced by situational factors.

The results also align conceptually with previous theoretical studies that introduced ternary reputation systems [41, 42], both of which demonstrated the evolutionary stability of cooperation under third-order norms within a ternary reputation framework. Our experimental results indicate that individuals adopt a more tolerant norm than that in the conventional ternary image scoring system [41]. Consistent with previous findings [42], the neutral evaluation appears to function as a buffer, allowing cooperation to stabilize more flexibly than in binary reputation systems. Furthermore, we demonstrate that individuals adopt a neutral stance toward justified defection and stabilize cooperation under a tolerant ternary reputation framework.

Our theoretical model based on evolutionary game theory has elucidated the mechanisms through which different action rules evolve and dominate the population in the gradating norm. Our analysis demonstrated that the system stabilizes a cooperative regime in the gradating norm. Additionally, the dominant behavioral rule was that of a tolerant discriminator, who only defects against individuals with bad reputations and cooperates with those with good or neutral reputations.

The results of our study provide valuable insights into the complexities of reputation dynamics and social norms in indirect reciprocity. Our findings reveal several critical patterns in how individuals evaluate and update reputations, challenging some of the prevailing assumptions in the literature. First, our experiment confirmed that reputational evaluations are not strictly binary but can be categorized into good, bad, and neutral. This nuanced classification reflects a more sophisticated understanding of reputation, aligning with recent research that suggests individuals perceive reputations with greater granularity [21]. The recognition of a neutral category alongside good and bad reputations provides a richer framework for understanding how people assess and react to others’ behaviors in real-world scenarios. Secondly, our findings also reveal a discrepancy between theoretical predictions and experimental observations regarding the updating rule of reputation. While theories propose that observers swiftly update a donor’s reputation as good or bad based on a single observation, our results indicate that individuals apply a gradual updating rule. Notably, we observe an intriguing result: individuals do not update their ratings in cases of justified defection. While a theoretical study suggests that people disregard all actions directed at recipients with bad reputations [43], our findings demonstrate that individuals perceive cooperation with bad recipients as a positive action.

The fact that justified defection is not considered good may have important implications for punishment in society. Punishment is recognized as a straightforward yet effective mechanism for sustaining cooperation in social dilemmas [44, 45]. In indirect reciprocity, justified defection serves as a punishment that removes free riders. The second-order norm, which can maintain cooperation robustly, has often been associated with Stern Judging [9–12] and Simple Standing [5, 13, 14], both of which evaluate justified defection as good. By evaluating justified defection positively, these norms encourage punitive actions against free riders, thereby supporting the maintenance of cooperation. Anthropological research has further suggested that early-developing cognitive mechanisms may naturally lead individuals to view justified defection as morally acceptable [46]. However, recent studies have shown that people tend to adopt a robustly neutral stance toward justified defection [21, 47]. This tendency reflects a psychological reluctance to assign moral approval to punitive behavior, even when such behavior is arguably warranted. Our findings are consistent with earlier work, which indicates that individuals who administer punishment, even when it is normatively justified, do not necessarily enjoy reputational benefits in human societies [48–52]. Further research is needed to investigate the cultural specificity, contextual sensitivity, and developmental trajectory of attitudes toward justified defection.

Several limitations of this study warrant careful consideration. First, we recruited participants from a Japanese crowdsourcing platform, resulting in a sample predominantly composed of Japanese adults. This raises the possibility that the social norms observed in this study may partly reflect cultural values specific to Japanese society [53]. To assess the generalizability of the tolerant ternary norm identified here, future research should include cross-cultural samples, particularly from Western contexts. Second, in the present version of the theoretical analysis, we do not introduce assessment errors, and addressing this limitation should be a focus of future analyses. In fact, a system without assessment errors may fail to converge to a unique stationary state. Introducing a small assessment error could help ensure convergence to a unique stationary state, irrespective of the initial conditions. Third, while aggregating reputation into three values is statistically reasonable (S1 Fig), the granularity lost through this aggregation needs to be further investigated. A computational approach, such as modeling reputation with more finely graded scales, could provide valuable insights into how more detailed reputation information influences social evaluations and cooperative behavior.

This study makes significant theoretical contributions by integrating higher-order information and multi-value reputation systems into the analysis of indirect reciprocity. Our results bridge the gap between experimental and theoretical approaches to reputation and social norm dynamics, offering a more comprehensive understanding of how cooperation can be sustained in complex social systems. Future research could explore the implications of these findings in real-world scenarios, where reputations are often multifaceted and influenced by a range of contextual factors.

## Methods

### Experimental settings

In the experiment, all 18 total conditions of three factors, donor’s reputation (good/bad/neutral), donor’s action (cooperation/defection), and recipient’s reputation (good/bad/neutral), were manipulated by using a between-subjects design. The experiment was conducted on 8th, November 2024, and a total of 1850 responses were collected. Participants were recruited using the website “Yahoo! Crowdsourcing” in Japan.

An example of one of the scenarios is as follows. The participants were assumed to be workers in a restaurant. Assume that a colleague, Bob (recipient), asks another colleague, Alice (Donor), to take over the night shift, and Alice agrees (cooperation) or refuses (defection). We also controlled Alice and Bob’s reputation. Below is an example of a good reputation donor defecting against a bad reputation recipient. For additional cases, refer to the Supplementary Information.

Alice works hard and is always willing to take over when others cannot come to do the night shift. That is why Alice is liked very much by colleagues in the restaurant including you. On the other hand, Bob is not serious about his work. Even when other employees ask him to cover for them on night shifts, he rarely agrees, even when he has the time. For this reason, Bob is not well thought of by colleagues in the restaurant including you. One day, Bob asked Alice to cover for him on the night shift because he wanted to go to a concert of his favorite singer. Although Alice had plenty of time, she declined Bob’s request.

After reading the scenario, participants rated how they assessed the donor’s behavior from three viewpoints using a 5-point scale: “Alice is a reliable person”, “Do you like Alice?”, and “Alice is approachable.” The evaluation scores for the donor’s action were added and normalized after checking the one-factor structure, and we used them as the participants’ evaluation scores for the donor. In the actual experiment, the names of the donor and recipient were converted into common Japanese names. A score of 0.0 means that the participant rated the donor’s behavior most negatively, and 1.0 means that the participant rated the donor’s behavior most positively.

### Ethics

The present series of experiments was approved by the Ethics Committee of Rissho University (Ethics approval number: 06-02) and conducted in accordance with the requirements of the Declaration of Helsinki. All participants were informed about the purpose of the study as well as the ways the data would be used. They agreed that the data would be used only for scientific research, that all data would be anonymized, and that they had the right to stop responding at any time. Informed consent was obtained from all participants.

### Fractions of reputation labels with each action rule

Let $d^{\prime }=(d,a,r)$ be the new reputation label $d^{\prime }\in R$, which is defined as the case where a donor with the reputation label d∈R acts a∈{0,1} toward a recipient with the reputation label r∈R. In the social norm discovered in our experimental study, $d^{\prime }=\min\{d+1,2\}$ when *a* = 1. If *a* = 0, $d^{\prime }=d$ when *r* = 0, and $d^{\prime }=\max\{0,d-1\}$ when *r*>0. We call the social norm “gradating” because $\max|d^{\prime }-d|=1$.

We calculate $g_{r}(a_{i}|p)$ defined above. The simultaneous equations for $g_{r}(a_{i}|p)$ are derived in Eq (3).


$$g_{0}(a_{i}|p)=g_{0}(a_{i}|p)\sum_{r\in R}D_{r}(a_{i}|p)+g_{1}(a_{i}|p)\sum_{r\in \{1,2\}}D_{r}(a_{i}|p)$$

$$g_{1}(a_{i}|p)=g_{0}(a_{i}|p)\sum_{r\in R}C_{r}(a_{i}|p)+g_{1}(a_{i}|p)D_{0}(a_{i}|p)+g_{2}(a_{i}|p)\sum_{r\in \{1,2\}}D_{r}(a_{i}|p)$$ (3)

$$g_{2}(a_{i}|p)=g_{1}(a_{i}|p)\sum_{r\in R}C_{r}(a_{i}|p)+g_{2}(a_{i}|p)[D_{0}(a_{i}|p)+\sum_{r\in R}C_{r}(a_{i}|p)],$$

where


$$D_{r}(a_{i}|p)=\sum_{a_{j}\in A}p_{j}g_{r}(a_{j}|p)(1-i_{r}+ei_{r}),\;C_{r}(a_{i}|p)=\sum_{a_{j}\in A}p_{j}g_{r}(a_{j}|p)i_{r}(1-e).$$

We explain the equation for $g_{1}(a_{i}|p)$ as an example. The first term of the equation is for the case that the player’s reputation label is 0 (its probability is $g_{0}(a_{i}|p)$). In that case, the player’s reputation label will be updated to 1 if and only if the player cooperates. If the reputation label of the player’s potential recipient is *r*, the probability the player cooperates is $i_{r}(1\;-\;e)$ (because when *i*_*r*_ = 1, the player cooperates if and only if the implementation error does not occur, and when *i*_*r*_ = 0, the player defects without errors) because one’s action rule is *i*_*r*_. The potential recipient’s action rule is *a*_*j*_ with the probability *p*_*j*_, and the reputation label is *r* with the probability $g_{r}(a_{j}|p)$. The second term of the definition is the case that the player’s reputation label is 1. In that case, the label keeps to 1 if and only if the player defects against someone with a 0 reputation label. If *i*_*r*_ = 1, which means that the player cooperates, the player defects when an implementation error occurs, and thus, the probability of defecting is *e*. If *i*_*r*_ = 0, which means that the player defects, the player absolutely defects. Therefore, the probability of defecting is integrated into $1\;-\;i_{r}\;+\;ei_{r}$. The explanation of the third term is omitted.

The 24 values expressed by $g_{r}(a_{i}|p)$ are determined by the 24 simultaneous equations defined above (note that $\sum_{r}g_{r}(a_{i}|p)=1$), but analysis is generally difficult. Therefore, we introduce discrete time. Calculating the right side of Eq (3) as the value at time *t* and treating the result as a difference equation for time t+1, the solution can be found by numerical simulations.

### ESS analysis in single population with no errors

We analyze a special case where all players adopt the same action rule with no error, i.e., let $g_{r}(a_{i})=g_{r}(a_{i}|p)$, where p=(0,0,…,1,…,0) (*p*_*i*_ = 1), and *e* = 0. Table 2 shows the values of *g*(*a*_*i*_) which satisfy Eq (3) for each action rule *a*_*i*_ in *A*.

**Table 2:** Values of *g*(*a*_*i*_) satisfying Eq (2) for each action rule in *A*

Action Rule	$g(a_{i})=(g_{0},g_{1},g_{2})$
*a*_0_	(1,0,0)
*a*_1_	(1,0,0)
*a*_2_	(1/3,1/3,1/3)
*a*_3_	(0,0,1)
*a*_4_	$\left(g^{*},\frac{(g^{*}){\;}^{2}}{1-g^{*}},\frac{(g^{*}){\;}^{3}}{(1-g^{*}){\;}^{2}})≒(0.43,0.32,0.25\right)$
*a*_5_	(0,0,1)
*a*_6_	$\left(\frac{(g^{*}){\;}^{3}}{(1-g^{*}){\;}^{2}},\frac{(g^{*}){\;}^{2}}{1-g^{*}},g^{*})≒(0.25,0.32,0.43\right)$
*a*_7_	(0,0,1)

In the cases of *a*_2_ and *a*_3_, there is another solution g=(1,0,0), but this solution has an unstable equilibrium, and we will show the proof in the case of *a*_3_. If *g*_0_(*t*)<1 then *g*_0_(*t*) decreases over time *t* because $g_{0}(t+1)=g_{0}(t){\;}^{2}$. Note that in the case of *a*_4_, the definition of *g*_0_ yields $g_{1}=\frac{(g_{0}){\;}^{2}}{1-g_{0}}$, and the definition of *g*_2_ yields $g_{2}=\frac{(g_{0}){\;}^{3}}{(1-g_{0}){\;}^{2}}$. Substituting them into $g_{0}+g_{1}+g_{2}=1$, $(g_{0}){\;}^{3}-2(g_{0}){\;}^{2}+3g_{0}-1=0$ is yielded. There is only one solution, $g_{0}=g^{*}$, of this cubic function in $0\leq g_{0}\leq 1$.

Next, we consider a reputation label of an *x* player (a player adopting the $x=[x_{0}x_{1}x_{2}]$ action rule) who invades a *y* population (a population consisting of players who all adopt the $y=[y_{0}y_{1}y_{2}]$ action rule), which is defined as S(x|y). Let $g_{r}^{*}(x|y)$, where r∈R is the fraction of the *r* reputation label given to the invader (*x*) in the case of S(x|y). The definitions of $g_{r}^{*}(x|y)$ are presented in Eq (4). Compared with the definitions of *g*_*r*_(*y*), all are the same except that the player’s reputation label is revised to $g_{r}^{*}(x|y)$ from *g*_*r*_(*y*). Thus, the definitions of $g_{r}^{*}(x|y)$ are


$$g_{0}^{*}(x|y)=g_{0}^{*}(x|y)\sum_{r\in R}D_{r}^{*}(x|y)+g_{1}^{*}(x|y)\sum_{r\in \{1,2\}}D_{r}^{*}(x|y)$$

$$g_{1}^{*}(x|y)=g_{0}^{*}(x|y)\sum_{r\in R}C_{r}^{*}(x|y)+g_{1}^{*}(x|y)D_{0}^{*}(x|y)+g_{2}^{*}(x|y)\sum_{r\in \{1,2\}}D_{r}^{*}(x|y)$$ (4)

$$g_{2}^{*}(x|y)=g_{1}^{*}(x|y)\sum_{r\in R}C_{r}^{*}(x|y)+g_{2}^{*}(x|y)[D_{0}^{*}(x|y)+\sum_{r\in R}C_{r}^{*}(x|y)],$$

where


$$D_{r}^{*}(x|y)=g_{r}(y)(1-x_{r}),\;C_{r}^{*}(x|y)=g_{r}(y)x_{r}.$$

We are ready to calculate their expected payoffs. In the case of S(x|y), the invader’s expected payoff, denoted as *P*_*x*_(*x*|*y*), and the resident’s expected payoff, denoted as *P*_*y*_(*x*|*y*), are defined as

$$P_{x}(x|y)=b\sum_{r\in R}y_{r}g_{r}^{*}(x|y)-c\sum_{r\in R}x_{r}g_{r}(y)$$ (5)

$$P_{y}(x|y)=(b-c)\sum_{r\in R}y_{r}g_{r}(y).$$ (6)

This is why a player with an *x* action rule invades a population of players with a *y* action rule if and only if $P_{x}(x|y)>P_{y}(x|y)$. We then define that an action rule *x* is evolutionarily stable if $P_{x}(x|y)\geq P_{y}(x|y)$, and $P_{x}(x|y)=P_{y}(x|y)\to P_{x}(y|x)>P_{y}(y|x)$ for, in the case of S(x|y), any y∈A, and y≠x.

Table 3 shows the expected payoffs of *P*_*x*_(*x*|*y*) in S(x|y). Note that in $S(a_{0}|a_{1})$, the equation system for $g_{r}^{*}(a_{0}|a_{1})$ does not determine any value, and thus, $P_{a_{0}}(a_{0}|a_{1})=bg_{2}^{*}(a_{0}|a_{1})$ for any $0\leq g_{2}^{*}(a_{0}|a_{1})\leq 1$, and thus, we set *b*/2 in that case. In Table 3, we use the values of $\left(g_{0}(a_{2}),g_{1}(a_{2}),g_{2}(a_{2}))=(1/3,1/3,1/3\right)$. The values in the cases of $y\in \{a_{4},a_{6}\}$ are approximated.

**Table 3:** Expected payoffs of *P*_*x*_(*x*|*y*) in S(x|y).

y	*x* = *a*_0_	*x* = *a*_1_	*x* = *a*_2_	*x* = *a*_3_	*x* = *a*_4_	*x* = *a*_5_	*x* = *a*_6_	*x* = *a*_7_
*a*_0_	0	0	0	0	–c	–c	–c	–c
*a*_1_	$\frac{b}{2}$	0	$\frac{b}{2}$	$\frac{b}{2}$	b–c	b–c	b–c	b–c
*a*_2_	0	$\frac{b-c}{3}$	$\frac{b-c}{3}$	$-\frac{2c}{3}$	$\frac{2b}{7}-\frac{c}{3}$	$\frac{2b}{7}-\frac{2c}{3}$	$\frac{2b}{7}-\frac{2c}{3}$	–c
*a*_3_	0	b–c	0	b–c	0	b–c	0	b–c
*a*_4_	b	0.43b–0.25c	0.25b–0.32c	–0.57c	0.43(b–c)	0.13b–0.68c	0.08b–0.75c	–c
*a*_5_	b	b–c	b	b–c	b	b–c	b	b–c
*a*_6_	b	0.57b–0.43c	0.75b–0.32c	–0.75c	0.92b–0.25c	0.41b–0.68c	0.57(b–c)	–c
*a*_7_	b	b–c	b	b–c	b	b–c	b	b–c

Therefore, we prove that no action rules are evolutionarily stable except for *a*_2_. Note that, when *a*_2_ forms the majority in the population, *a*_1_ can invade isolatedly because $P_{a_{1}}(a_{1}|a_{2})=P_{a_{2}}(a_{2}|a_{2})$. In the situation $S(a_{2}|a_{1})$, $P_{a_{2}}>P_{a_{1}}$, so a different strategy is required for *a*_1_ to become the majority. The key point is a situation in which *a*_2_ is the majority in the population, but *a*_1_, who has gained citizenship as a neutral mutant, is present at a certain rate. In this situation, *a*_3_ can invade because it can increase expected payoffs in the *a*_1_ world. This is possible if mutants always invade. If there is a regime of *a*_3_, both *a*_1_ and *a*_3_ can invade rapidly. This effect first allows the hegemony of the population to shift from *a*_2_ to *a*_1_. Then, this regime change promotes the rapid proliferation of *a*_3_, and like a phase transition, the *a*_2_ hegemony era will be replaced by the *a*_3_ hegemony era. This will realize a stable cooperative regime. When *a*_3_ forms the majority of the population, *a*_5_ and *a*_7_ (unconditional cooperators) can invade as neutral mutants (hence, *a*_3_ is not an ESS). However, they are driven out by *a*_0_ (unconditional traitors) who always invade in small numbers due to the effect of random drift, so only a small number can survive in the population at any one time.

### Robustness check on the performance of the initial population

In Results, we analyzed whether there exists an action rule with evolutionary stability that satisfies a high cooperation rate in order to show the theoretical possibility of the social norm referred as gradating. For this reason, it is not necessary to show that the discovered action rule *a*_3_ can invade any initial population. However, since the model we used for our analysis always has new invaders by mutation, no strategy can become 0, so it is clear that it can only have stable points inside the simplex dynamically. Empirically, this makes it easier to eliminate unstable equilibrium points occurring at the boundary and to reach a stable resting point. For these reasons, although we did not provide a rigorous proof in this paper due to analytical difficulties, the action rule *a*_3_ discovered this time is highly likely to have global stability. To demonstrate this point, we additionally confirmed that even if the simulation was started from a different initial population, *a*_3_ eventually forms a population as the majority. The specific simulation method is as follows. Parameters are set to (b,c,e,μ)=(3,1,3%,3%). An initial population consisting of a single action rule is set for each action rule. In this case, eight simulations are performed. In all of these, *a*_3_ was confirmed to be the final winner. Each simulation shows that *a*_3_ is reached even when a population generated during the simulation is used as the initial population, suggesting that *a*_3_ is highly likely to have global asymptotic stability dynamically.

## Supporting information

S1 File This file includes descriptions of experimental scenarios and statistical analyses, including S1–S4 Tables and S1–S3 Figs. (DOCX)
