# Supplementary material for: Gradual reputation dynamics evolve and sustain cooperation in indirect reciprocity
Source: PLoS One. 2025 Aug 8;20(8):e0329742. doi: 10.1371/journal.pone.0329742 (PMC12334055; doi:10.1371/journal.pone.0329742)
Supplement: S1 File — (DOCX) [file pone.0329742.s001.docx]

**Supporting Information** for

Gradual reputation dynamics evolve and sustain cooperation in indirect reciprocity

Hitoshi Yamamoto^1*^, Isamu Okada^2*^, and Takahisa Suzuki^3^

1: Rissho University, JAPAN, 2: Soka University, 3: Tsuda University, *: These authors contributed equally.

Material of Experiments

First, the two characters (donor and recipient) are presented as colleagues at the restaurant in addition to the participants. Then, the reputations of the donor and the recipient are controlled, and the donor takes cooperative/non-cooperative actions. Finally, the participants evaluate their impressions of the donor.

**Introduction of scenario**

Consider that you are working in a restaurant. Alice and Bob are co-workers. In this restaurant, employees are assigned to work the night shift. Each has a habit of asking other employees to take their place on the night shift when they are not available.

The following shows the characters’ good and bad reputations in the scenario. In the case of a neutral reputation, all descriptions of the characters’ reputations have not been shown. We combined these to control the reputations of the donors and recipients. In the actual experiment, the names of the donor and recipient were converted into common Japanese names.

**Good reputation example**

Alice works hard and is always willing to take over when others cannot come to do the night shift. That is why Alice is liked very much by colleagues in the restaurant including you.

**Bad reputation example**

Bob is not serious about his work. Even when other employees ask him to cover for them on night shifts, he rarely agrees, even when he has the time. For this reason, Bob is not well thought of by colleagues in the restaurant including you.

We presented descriptions of the reputations of the donors and recipients and then presented the behaviour of the donors.

**Cooperation**

One day, the recipient asked the donor to cover for her/him on the night shift because she/he wanted to go to a concert of her/his favorite singer. The donor was very tired from working night shifts, but he/she accepted the recipient’s request.

**Defection**

One day, the recipient asked the donor to cover for her/him on the night shift because she/he wanted to go to a concert of her/his favorite singer. Although the donor had plenty of time, she/he declined the recipient’s request.

After reading the scenario, participants rated how they assessed the donor’s behavior from three viewpoints using a 5-point scale: “The donor is a reliable person,” “Do you like the donor?”, and “The donor is approachable.” In the actual experiment, the names of the donor and recipient were converted into common Japanese names. The evaluation scores for the donor’s action were obtained by simply adding together the scores of these three statements. When the three items were summed, the total ranged from 3 to 15. The values were then normalized to fit within the range of 0 to 1.

Statistical analysis for experiment

**Table S1. Descriptive statistics for experiment:** Each value represents mean, variance, and skewness of evaluation of donor’s behavior in each of 18 scenes.

| **Action (C/D)** | **Who (Donor)** | **To whom (Recipient)** | **N** | **mean** | **variance** | **skewness** | **alpha** |
| --- | --- | --- | --- | --- | --- | --- | --- |
| D | B | B | 104 | 0.204 | 0.051 | 0.945 | 0.938 |
|  |  | G | 112 | 0.14 | 0.041 | 1.762 | 0.878 |
|  |  | N | 107 | 0.15 | 0.043 | 1.124 | 0.929 |
|  | N | B | 101 | 0.54 | 0.052 | -0.334 | 0.883 |
|  |  | G | 105 | 0.298 | 0.076 | 0.687 | 0.95 |
|  |  | N | 104 | 0.346 | 0.058 | 0.356 | 0.911 |
|  | G | B | 106 | 0.681 | 0.048 | -0.576 | 0.878 |
|  |  | G | 101 | 0.496 | 0.064 | 0.048 | 0.923 |
|  |  | N | 104 | 0.59 | 0.055 | -0.14 | 0.916 |
| C | B | B | 96 | 0.487 | 0.066 | -0.165 | 0.916 |
|  |  | G | 94 | 0.532 | 0.056 | -0.427 | 0.905 |
|  |  | N | 106 | 0.542 | 0.05 | -0.423 | 0.871 |
|  | N | B | 101 | 0.744 | 0.061 | -1.325 | 0.914 |
|  |  | G | 101 | 0.832 | 0.027 | -0.889 | 0.842 |
|  |  | N | 100 | 0.796 | 0.031 | -1.078 | 0.844 |
|  |  | B | 105 | 0.767 | 0.049 | -0.98 | 0.83 |
|  |  | G | 101 | 0.83 | 0.027 | -0.719 | 0.871 |
|  |  | N | 102 | 0.797 | 0.036 | -0.685 | 0.863 |

**Figure S1: Cluster analysis for experiment:** Panel A illustrates Within-cluster sum of squares (WSS), indicating that three clusters provide best fit. Panel B presents dendrogram from cluster analysis.


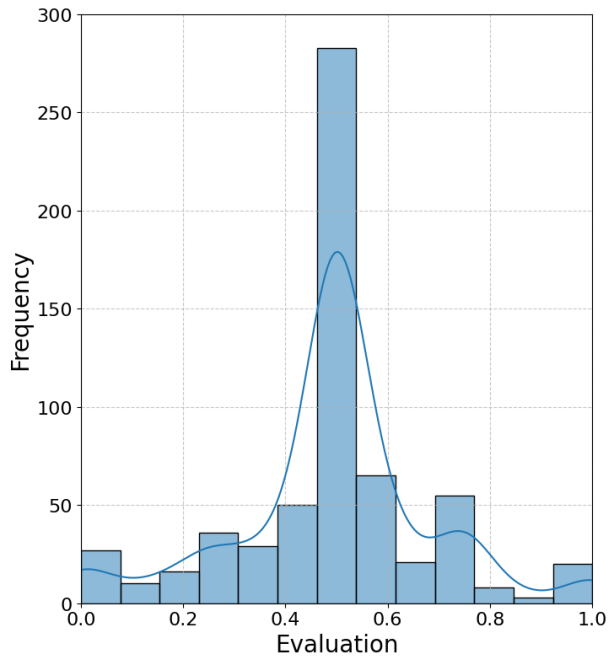


**Figure S2: Histogram of the participants' evaluations of recipients without reputation information:** We summarized six scenes of evaluation in which the donor (good, neutral, bad) acts (cooperates, defects) towards the neutral recipient.

**Table S2: Best-fitting distribution for each distribution based on Bayesian Information Criterion (BIC):** Each value represents BIC, with smallest value indicating best fit. Smallest value for each fit is highlighted in bold.

| **Who acts toward whom** | **Exponential dist.**  **(Bad evaluation)** | **Normal dist.**  **(Neutral evaluation)** | **Reflected exponential dist.**  **(Good evaluation)** |
| --- | --- | --- | --- |
| BDB | **399.194** | 511.517 | 681.9631 |
| BDG | **344.738** | 526.0109 | 751.5843 |
| BDN | **344.903** | 507.8178 | 715.5854 |
| NDG | **481.977** | 557.296 | 662.2957 |
| NDN | **508.844** | 523.348 | 641.1293 |
| BCB | 535.5159 | **497.336** | 545.5182 |
| GDG | 566.877 | **518.413** | 570.2104 |
| GDN | 619.6669 | **519.355** | 544.1826 |
| BCG | 541.0267 | **471.091** | 516.994 |
| BCN | 613.4855 | **517.898** | 578.07 |
| NDB | 583.9485 | **498.785** | 551.8813 |
| GDB | 661.958 | 513.0623 | **501.361** |
| NCN | 655.9134 | 441.3288 | **383.823** |
| GCG | 670.9357 | 433.2995 | **350.592** |
| NCG | 671.3369 | 433.8414 | **348.621** |
| NCB | 648.8929 | 514.3302 | **433.148** |
| GCB | 680.904 | 511.9465 | **430.159** |
| GCN | 669.3569 | 465.4473 | **389.87** |


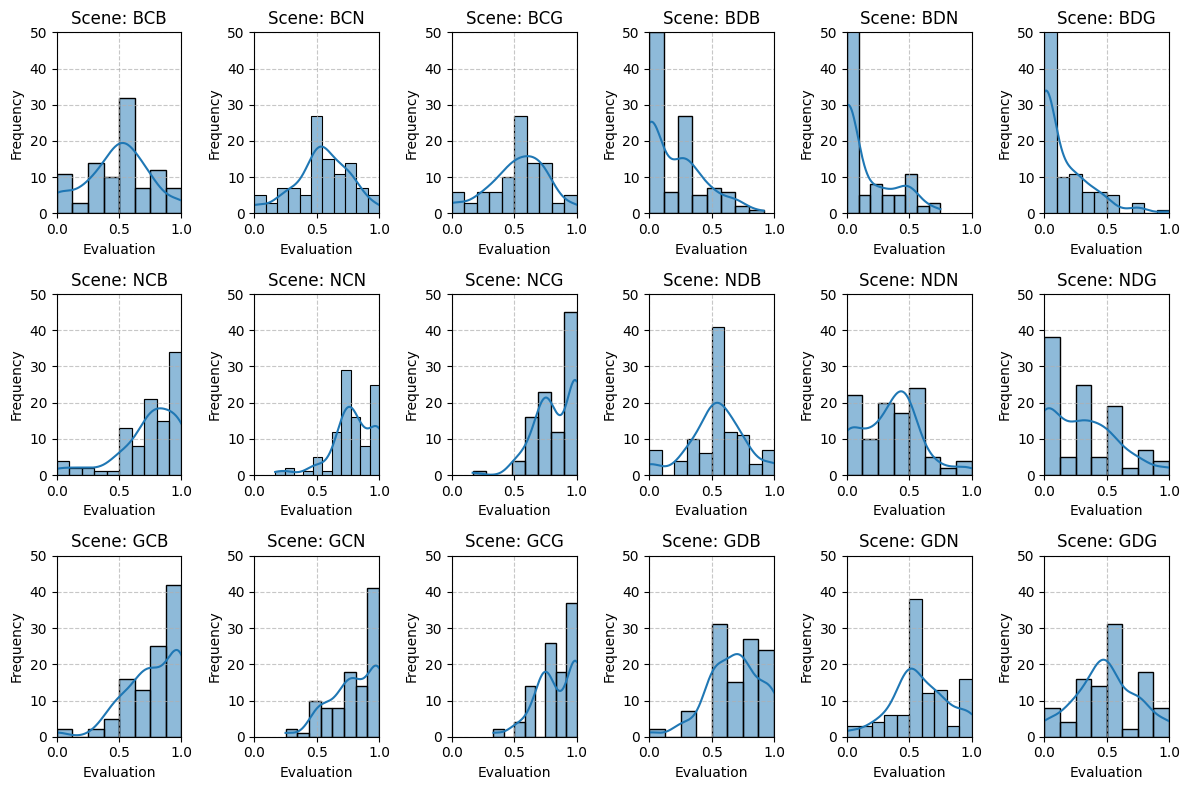


**Figure S3: Histogram of the 18 scenes in the Evaluation:** The curve in each panel represents the kernel density estimation.

As part of the supplementary analysis, we tested the differences in the mean evaluations across all 18 scenes. To achieve this, we used the Kruskal-Wallis test as a non-parametric method for comparing multiple groups. Since the test revealed a significant difference (H(17) = 953.427, p < .001), we conducted a Dunn test with Bonferroni correction for multiple comparisons. Table S3 presents the p-values for each combination. While some combinations of NDN and GDB showed no significant differences between the Neutral, Bad, and Good categories or significant differences within categories, the overall findings are consistent with the cluster analysis results.

**Table S3: The p-values for each combination of the Dunn test:** Cases with no significant difference between categories and cases with significant differences within categories are highlighted in bold.

**Table S4. Examination of gender differences in donor's evaluation:** This table shows the results of t-tests for gender differences in 18 scenes. Although the differences in donor evaluations for DBN and DBB were statistically significant, both conditions were still categorized as Bad.

| action, donor, recipient | N (male) | N (female) | mean (male) | mean (female) | t-value | p-value |
| --- | --- | --- | --- | --- | --- | --- |
| DGG | 53 | 48 | 0.520 | 0.469 | 1.030 | 0.306 |
| DGN | 70 | 34 | 0.580 | 0.610 | -0.619 | 0.537 |
| DGB | 65 | 41 | 0.691 | 0.665 | 0.605 | 0.547 |
| DNG | 73 | 32 | 0.304 | 0.284 | 0.338 | 0.736 |
| DNN | 66 | 38 | 0.331 | 0.373 | -0.859 | 0.393 |
| DNB | 62 | 39 | 0.513 | 0.581 | -1.458 | 0.148 |
| DBG | 61 | 51 | 0.161 | 0.114 | 1.217 | 0.226 |
| DBN | 69 | 38 | 0.181 | 0.094 | 2.099 | **0.038** |
| DBB | 67 | 37 | 0.164 | 0.277 | -2.492 | **0.014** |
| CGG | 56 | 45 | 0.841 | 0.817 | 0.726 | 0.469 |
| CGN | 65 | 37 | 0.814 | 0.768 | 1.182 | 0.240 |
| CGB | 59 | 46 | 0.754 | 0.784 | -0.690 | 0.492 |
| CNG | 61 | 40 | 0.814 | 0.858 | -1.313 | 0.192 |
| CNN | 64 | 36 | 0.786 | 0.813 | -0.709 | 0.480 |
| CNB | 65 | 36 | 0.765 | 0.706 | 1.159 | 0.249 |
| CBG | 68 | 26 | 0.526 | 0.548 | -0.408 | 0.685 |
| CBN | 67 | 39 | 0.544 | 0.538 | 0.112 | 0.911 |
| CBB | 60 | 36 | 0.469 | 0.516 | -0.859 | 0.392 |
